# Patients’ Perspective on the Value of Medication Management Appointments

**DOI:** 10.3390/healthcare3020284

**Published:** 2015-05-20

**Authors:** Mario Cruz, Robyn Flaum Cruz, Harold Alan Pincus

**Affiliations:** 1 Department of Psychiatry, University of New Mexico, 2600 Marble Avenue NE, Albuquerque, NM 87106, USA; 2 Graduate School of Arts and Social Sciences, Division of Expressive Therapies, Lesley University, Cambridge, MA 02138, USA; E-Mail: rcruz@lesley.edu; 3 Department of Psychiatry, Columbia University School of Medicine, New York, NY 10032, USA; E-Mail: pincush@nyspi.columbia.edu

**Keywords:** appointment value, depression, appointment adherence

## Abstract

*Objectives*: There is ongoing concern that psychiatric medication management appointments add little value to care. The present study attempted to address this concern by capturing depressed patients’ views and opinions about the value of psychiatric medication management appointments. *Methods*: Seventy-eight semi-structured interviews were performed with white and African American depressed patients post medication management appointments. These interviews tapped patients’ views and opinions about the value of attending medication management appointments. *Analysis*: An iterative thematic analysis was performed. Findings: Patients reported greater appointment value when appointments included obtaining medications, discussing the need for medication changes or dose adjustments, and discussing the impact of medications on their illness. Additionally, greater appointment value was perceived by patients when there were non-medical conversations about life issues, immediate outcomes from the appointment such as motivation to continue in care, and specific qualities of providers that were appealing to patients. *Conclusions*: Patients’ perceived value of psychiatric medication management appointments is complex. Though important patient outcomes are obtaining medicine and perceiving improvement in their mental health, there are other valued appointment and provider factors. Some of these other valued factors embedded within medication management appointments could have therapeutic properties. These findings have implications for future clinical research and service delivery.

## 1. Introduction

Medication management [1] appointments are brief, spread out over time [2,3,4,5], and create the principle context within which psychiatrists provide outpatient care. In this setting, psychiatrists provide pharmacotherapy.

Kontos and colleagues [6] view pharmacotherapy as specific, narrow, and explicitly “medical” rather than “psychotherapeutic”. Although the pharmacotherapist’s role in care has been well-described, there is concern the role is insufficient to address patients’ psychosocial needs [7]. In addition, other investigators have expressed concern that the pharmacotherapeutic relationship may “disengage” the psychiatrist from exploring issues important to care delivery. Some of these issues are educating the patient about the nature of his/her illness [8], negotiating a treatment plan [9], developing a trusting, caring, and participatory relationship with the patient [8], and activating patient self-management skills [6].

Contrary to the above mentioned concerns, our previous communication research findings suggest that psychiatrists devote a significant portion of their verbal communication behaviors in medication management appointments to non-pharmacotherapy activities that are core features of patient-centered care [10,11,12,13]. Patient-centered activities include providing illness and treatment option information; partnering with patients to negotiate a treatment plan; building rapport through conveying warmth, empathy, and caring; activating treatment adherence behaviors; and asking about as well as counseling patients regarding psychosocial and lifestyle issues [14,15] In addition, we found future appointment adherence, an appointment outcome that has been positively related to medication adherence and negatively related to hospitalization rate, was not related to psychiatrist verbal communication behaviors [3,16,17,18,19,20].

We postulated that the negative verbal communication behavior finding may have been the consequence of a limited understanding on our part of what features of medication management appointments patients find valuable or worthwhile. The present study intended to address this gap in our understanding of medication management appointments by capturing patient perceptions of what is valuable within medication management appointments.

## 2. Methods

### 2.1. Study Procedures

Three interviewers performed 10 to 15 min semi-structured interviews post medication management appointments at four ambulatory mental health clinics within a large, urban university-affiliated mental health care system in the Midwest. The interviews tapped patients’ views and opinions regarding the value of the just completed medication management appointment.

Patients were eligible if they were 18–65 years of age, had a chart recorded diagnosis of major depressive disorder, depressive disorder-nos, or dysthymic disorder as defined in the Diagnostic Statistical Manual Fourth Edition, revised (DSMI-IV TR), and if they were in treatment with a participating psychiatrist.

Patients were either self-referred in response to a flyer or were introduced to the study by their therapist or psychiatrist. Patient participants received a $10.00 grocery store gift card for study completion. Psychiatrists received no compensation.

The study was approved by the University of Pittsburgh Institutional Review Board and all participants signed an approved informed consent form.

### 2.2. Clinic Recruitment

We recruited three community-based and one mood disorder research clinic (see Table 1 for details). The psychiatrists’ role in the participating sites was limited to pharmacotherapy in split treatment appointments. At all clinics, psychotherapy was provided by Master’s level clinicians.

Two clinics provided the majority of study participants (N = 67, 74%). Clinic 1 was staffed with both attending and resident psychiatrists. Clinic 2 was staffed by resident psychiatrists under the supervision of an attending psychiatrist. The additional two clinics were staffed by attending psychiatrists.

**Table 1:** Patient Demographic Characteristics.

Patients		
Age, M (range)	45.0 (24–65)	**
	*N*	*%*
Gender		
Female	65	83.3
Male	13	16.7
Race		
White	34	43.6
Black	44	56.4
Marital Status		
Married	13	16.7
Unmarried	65	83.3
Income Status		
<10,000	39	50.0
10,000–39,900	19	24.4
≥40,000	20	25.6
Employment Status		
Full-time Employed	14	18.0
Part-time Employed	5	6.4
Homemaker	5	6.4
Retired	1	1.3
Unemployed	12	15.4
Disabled	41	52.5
Insurance Status		
Public	43	55.1
Private	17	21.8
None	18	23.1
Age, M (range)	45.0 (24–65)	**
	*N*	*%*
Education		
<HS	13	16.7
HS/GED	28	35.9
Post-Secondary	37	47.4
Clinics		
1	18	23.0
2	39	50.0
3	13	16.7
4	8	10.3

### 2.3. Data Collection

Prior to the patient’s medication management appointment, research staff met with the patient, obtained written, informed consent, and collected patient demographic information as well as chart recorded psychiatric diagnoses. Immediately following the appointment, the patient met with the study’s research staff in a private office to complete the semi-structured interview.

To begin the interview, patients were primed to think of their appointment experience by having them rate the appointment value compared to other things they could have been doing. Value ratings were scored on a 0–3 Likert type scale with 0 = not valuable at all and 3 = very valuable. After completion of the value rating question, patients were asked, “What about the appointment made you rate its value as you did?”

The interview combined open and closed-ended questions to obtain as exhaustive a response to the value question. This use of open and closed-ended questions has been reported to be an ideal mix for situations when there is one chance to speak with a respondent [21]. The interviewer transcribed verbatim patient responses.

## 3. Data Analysis

The goal of this qualitative study was to describe the range of themes that patient participants perceived as influencing their value rating of medication management appointments. The first two authors developed the analytic strategy, acted as coders, and performed all elements of this analysis. Both authors have formal training in and experience with qualitative research. We employed two layers of coding as espoused by Glaser and Strauss [22]. In the first coding layer, the coders agreed that patient statements conveying one thought would be construed as analytic quotes or concepts. Thereafter, the coders independently reviewed all transcripts and looked for quotes that suggested processes, actions, assumptions, and consequences [22,23]. They also looked for metaphors and repetitions across participants that may indicate relevant themes [24]. After independently examining the transcripts and identifying quotes, the coders checked for agreement. If agreement was less than 100%, the coders met until all disagreements were resolved.

For the second layer of coding into larger theoretical categories, the coders used the cutting-and-sorting method of qualitative data analysis to group quotes into value related themes and sub-themes [25]. Quotes were cut from paper copies of transcripts. Reference information (*i.e.*, study ID, value score) was placed on the back of each paper quote cutout. Thereafter, all paper quote cutouts were randomly spread out on a table. Together, the coders sorted quotes into groups. After all groups of quotes were sorted, the coders examined all quotes within each group. Quote examination occurred in two stages. In the first stage, quotes were examined independently by the coders over several weeks. In the second stage, the coders met and sorted group quotes into quotes that were considered central to or essential features of and quotes that were peripheral to the group themes. Central quotes were then assessed to identify quotes that represented distinct sub-themes. Thereafter, themes and sub-themes were then given distinguishing names and definitions. Each quote was assigned to a single thematic and sub-thematic group. Exemplar quotes in each thematic and sub-thematic group were identified and are presented within the results section of this manuscript.

## 4. Results

### 4.1. Patient Recruitment

Of the 150 patients approached, 130 (86%) signed consent and 89 of the consenting patients (68.5%) provided responses to the value question. Of the 89 patient respondents to the value question, 11 patients’ responses were excluded from the analysis because their responses were brief statements that offered no clear insight as to their perceived appointment value (e.g., “No”; “Can’t say”; “Yes”). The final analysis was conducted on 78 (59% of consented patient sample) participant responses.

#### 4.1.1. Patient Participants’ Demographic Information (See Table 1)

On average, patient participants were 45 years of age (range 24–65), African American (N = 44, 57.1%), female (N = 64, 83.1%), unmarried (N = 64, 83.1%), had income below $20,000 per year (N = 61, 79.2%), and had an education level of high school graduate or above (N = 61, 79.2%).

#### 4.1.2. Patient Value Ratings

The range of value ratings was from 0 to 3. The majority of participants rated the value of their appointment a “3” (70.5%) while 15.4% of participants rated their appointment value a “2”, 12.8% rated their appointment value a “1” and 1.3% rated their appointment value a “0”.

#### 4.1.3. Thematic Analysis

**Figure 1:**
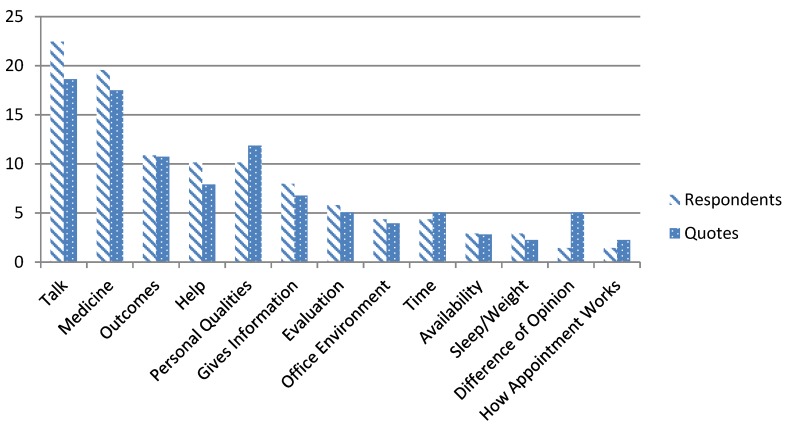
All value themes: percent of total respondents *versus* percent of total quotes.

Our analysis identified thirteen themes. Figure 1 shows proportions of respondents and quotes by theme. There were no significant differences between the proportions of respondents relative to the number of quotes per theme. Therefore, the majority of quotes within themes were expressed by more than one patient participant.

Sub-themes were identified for two thematic groups, *i.e.*, talk and medicine. The talk theme had two sub-themes (*i.e.*, one-way (provider listen) and two-way (discussion or counseling) conversations). The medicine theme had three sub-themes (*i.e.*, obtaining and adjusting medications, talking about medications, and effects of medications).

### 4.2. Talk

The most frequently occurring theme was talking with the psychiatrist. The “talk” could either be in the form of a one-way conversation where the psychiatrist would listen to the patient talk about issues important to them; or a two-way conversation where there was an interpersonal exchange regarding a particular topic the patient found important to discuss or to obtain advice on how to address. Strengthening the position that talk was valuable to patients, another patient who rated the value of the appointment a “0” reported the lack of talk devalued the appointment, “I think it would be helpful to talk more.”

The listen sub-theme issues were often related to present life difficulties or problems. In addition, patients who perceived one-way conversations as valuable found them therapeutic. Statements such as “Get frustration loose” or “Needed to get things off my chest” or “I am going through a financial crisis and I need to get it off my chest” strongly suggested there was a therapeutic benefit to patients being heard. One patient identified the non-judgmental stance of the psychiatrist as a reason for one-way conversations being valuable, “I can speak here and not be judged.”

The two-way conversation sub-theme was defined as a verbal exchange between the psychiatrist and patient that was either intended to be a discussion of a particular event where both parties are active participants in the conversation or some form of therapeutic intervention was offered, *i.e.*, counseling. Examples are, “Wanted to ask questions about meds and side effects,” “I was able to discuss concerns about my lower back and primary care provider’s prescription.” One patient highlighted the potential therapeutic benefit of a two-way conversation as, “Because it helped me with some of the issues I was having.”

### 4.3. Medicine

Patients perceived one core feature of medication management appointments is to obtain (“Put me on more of the medicine I take”), adjust (“Getting my medication straightened out”), or change medications (“I thought she might give me some sleep medicine”).

In addition, patients were also interested in discussing the effects, both present and future, of medications. For instance, one patient stated, “They helping me to be able to sleep.” Another patient expressed hopefulness regarding the medication she was being started on by stating, “I am counting on the medication helping me.”

### 4.4. Appointment Outcomes

Patients also felt that specific appointment outcomes were valuable. For example, one patient stated it was valuable to be reassured treatment was progressing well (“Reassurance that everything is OK”).

Some participants also reported that coming to appointments helped them stay motivated for treatment. For instance, one participant stated “It encourages me to not backslide or get complacent.” Another participant stated “Because it helps me to be concerned about myself.”

Other participants reported that coming to appointments helped them “To just stand the day,” or provided them with some protection from adverse outcomes. One participant said, “If I go to an appointment, I don’t have an episode,” while another reported “It keeps me from thinking violent things.”

The appointment was valued poorly if the patient felt he or she did not take anything away from the appointment. For example, one participant rated the value of his appointment a “0” and stated “There was really nothing done.”

### 4.5. Help

The patient’s desire to receive help was seen as an essential element to the perceived value of medication management appointments. For example, seeking help by coming to appointments and letting the psychiatrist know “how they are doing” were viewed as the two main patient behaviors that contributed to the value of appointments.

Participants also felt it was important to keep their psychiatrist informed as to their response to medications (“Because we talked about how my new medication was working,” “I need to keep my psych doc informed on how my meds are doing”) as well as their medication adherence (“Had to explain to the doctor I wasn’t taking medicine,” “What ones I don’t need to take anymore”). 

### 4.6. Personal Qualities

Several participants reported specific provider qualities that improved the value of appointments. We defined personal qualities as specific characteristics of the doctor that influence perceptions of appointment value. For example, one participant reported that feeling understood by her provider was valuable when she stated, “I think he (psychiatrist) understands me.” Another participant reported having a psychiatrist she can have a good relationship with was also valuable when she said, “Dr. F and I have a good relationship.” This quality was also supported by one patient who rated the appointment value as “1” and stated “I don’t get to have much of a relationship with my psychiatrist.”

Other valuable qualities identified were providers not being closed minded, patients feeling something is being done about their problem, and having a psychiatrist who can listen and not “rush you out” of the appointment.

### 4.7. Availability

Having a provider who could be contacted outside of appointment times was also valuable. One patient recalled, “When I was depressed in June 2006, he said call me quickly so I can see you earlier.” She reported the value of availability as, “I think you need to know somebody is there.”

### 4.8. Gives Information

Counseling and patient education were also viewed as valuable elements of medication management appointments. One participant stated “He provides helpful solutions.” Another participant reported scoring the appointment a “3” for “Getting info or facts I can use. Things I can do about the way I’m feeling.” In contrast, another participant had scored the appointment a “1” and said the reason was, “There are some mental health issues that are not explained so I can understand them.”

### 4.9. Evaluation

An essential feature of these appointments was having the psychiatrist evaluate their present mental status. One participant put it this way, “Just for him to see something maybe I don’t see.” Another participant reported, “Just like a medical doctor, I need to know how well I’m doing.”

### 4.10. How Appointment Works and Welcoming Environment

Some participants noted that organizational features of the clinic they attend influenced their perceived appointment value. One organizational feature seen as valuable was orienting patients about service delivery. One participant rated the appointment value a “1” for, “I am still not familiar with how these visits work and what I should expect.” 

Several patients noted how they were treated by office staff and the ambience of the office environment. One participant noted, “Everybody down here treats me with respect.” Another participant stated, “I feel like it’s a warm environment.” 

Last, staff working as a team was also seen as valuable. One participant who rated the appointment value a “3” reported, “They work as a team to help solve a problem to help the patient.”

### 4.11. Time

Patients also saw appointment length and frequency as important factors in their value ratings of appointments. One participant who rated the appointment value a “1” commented, “Don’t have time to talk with him.” Another participant who rated the appointment value a “3” reported, “He took the time to explain everything.” One participant rated the appointment value a “2” and stated, “I would like to see this doctor more so she could know what’s going on.”

### 4.12. Sleep/Weight

Patients specifically reported improving their sleep and reducing their weight were important outcomes of care. One patient who rated the appointment a “3” said, “Because I need the help. They helping me to be able to sleep. Just stand the day.” Another patient reported “I wished to see the doctor too because I thought she might get me some sleep medicine.” In terms of weight reduction discussions, patients rated the value of the appointment a “3” when patients and providers actively discussed this as an important mental health care concern. For instance, one patient reported, “I have problems with my weight and we’re discussing how to cut down my eating habits and change my medications.” Another patient reported, “I was able to discuss concerns about my lower back and primary care provider’s medication, and my desire for weight loss. 

### 4.13. Difference of Opinion

Patients reported that when there was a difference of opinion regarding treatment the appointment value was decreased. As one participant said, “I’m aggravated that they are not giving me the medication I feel I need.”

## 5. Discussion

Our qualitative analysis revealed several appointment value-related themes. These themes encompassed issues associated with psychiatrists’ communication behaviors and their ability to develop trusting relationships with their patients, patient care access and appointment behaviors, and organizational features that could influence the perceived value of appointments.

What we found interesting about the participant quotes are that some quotes were focused on addressing the therapeutic impact of medications plus appointments over time such as patients finding, obtaining, adjusting, and discussing medications as well as patients’ requests for sleep issues related to their depressive illness and weight concerns secondary to unwanted antidepressant medication effects to be addressed.

Other quotes suggested a clear and immediate impact of features of the appointment that could be factors integral to treatment outcome such as maintaining treatment motivation, enhancing self-awareness, stopping violent thoughts, instilling hope that their condition will improve, and having someone listen to or discuss with them life-related issues. These appointment features could be critical therapeutic elements embedded within medication management appointments.

The study findings suggest the following recommendations to improve clinical practice. First, providers should devote a portion of the appointment time to allow patients to talk about their lives. In addition, our findings emphasize the importance practicing psychiatrists should place on exploring issues related to care delivery such as orienting patients to the role played by different behavioral health professionals in their care, reassuring patients about their care, giving patients feedback on their mental status, assessing and enhancing patients level of motivation to continue in care, counseling and educating patients about their illness and medications, and being responsive to patient’s needs outside of appointments. Our findings also suggest that behavioral health organizations and their administrators can improve the value of medication management appointments by considering the importance the clinic environment has on patient perceptions of care and allotting enough time to medication management appointments so psychiatrists can allow patients to talk about their lives, address care delivery issues, and to perform the tasks related to medication management.

This study has limitations. First, our directions for the value rating tool may have limited patients’ perceptions of what could be valuable within appointments. For instance, our directions asked participants to explain why they rated the appointment value a certain amount. Because their responses were bounded by their perceptions of an appointment *versus* their perceptions of medication management appointments in general, we may have lost other important value themes. Another study limitation is that our Likert scale may have been too limited for patients to express a nuanced synthesis of what is experienced as valuable in appointments. Last, our study attempted to address patient’s perceptions of medication management appointments divorced from their perceptions of the other component of treatment (therapy). This may have been difficult to do for some or all participants. Therefore, we could hypothesize that a participants desire to talk with their psychiatrist is influenced by the perceived value of their counseling appointments. Future studies should include capturing patients’ perceptions of the value of therapy appointments.

## 6. Conclusions 

In conclusion, this qualitative analysis of semi-structured patient participant interviews post-medication management appointments identified features of these appointments the participants found valuable compared to other things they could have been doing in their lives. Some of the non-specific elements embedded within medication management appointments, e.g., talking with patient’s about issues in their life may have therapeutic effects. Future quantitative studies could assess if these non-specific elements contribute to depression care outcomes.
